# The broad spectrum of cardiotoxicities from immunotherapies

**DOI:** 10.3389/fcvm.2023.1259620

**Published:** 2023-09-15

**Authors:** Martina Iengo, Ester Topa, Alessandra Cuomo, Giancarlo Marone, Remo Poto, Gilda Varricchi, Leonardo Cristinziano, Maria Rosaria Galdiero, Anne Lise Ferrara, Stefania Loffredo, Luigi Formisano, Teresa Troiani, Valentina Mercurio, Carlo Gabriele Tocchetti

**Affiliations:** ^1^ Department of Translational Medical Sciences, Federico II University, Naples, Italy; ^2^ Department of Pharmacy, Moscati Hospital Pharmacy, Aversa, Italy; ^3^ World Allergy Organization (WAO) Center of Excellence, Naples, Italy; ^4^ Center for Basic and Clinical Immunology Research (CISI), Federico II University, Naples, Italy; ^5^ Institute of Experimental Endocrinology and Oncology (IEOS), National Research Council, Naples, Italy; ^6^ Department of Medicine and Surgery, Federico II University, Naples, Italy; ^7^ Interdepartmental Center of Clinical and Translational Sciences (CIRCET), Federico II University, Naples, Italy; ^8^ Medical Oncology, Department of Precision Medicine, University of Campania Luigi Vanvitelli, Naples, Italy; ^9^ Interdepartmental Hypertension Research Center (CIRIAPA), Federico II University, Naples, Italy

**Keywords:** cardio-oncology, immunotherapy, cardiotoxicity, detection, management

## Introduction

Cancer immunotherapies have revolutionized antineoplastic treatments. CTLA-4, PD-1, and PD-L1 are crucial regulators of the immune response and play a central role in the maintenance of self-tolerance (1). Monoclonal antibodies directed against CTLA-4, PD-1, and PD-L1 block these immune checkpoints and unleash anti-tumor immunity, leading to tumor cell death through cytolytic molecules. Unfortunately, these immune checkpoint inhibitors (ICIs) either alone or in combination, can lead to imbalances in immunologic tolerance resulting in a broad spectrum of immune-related adverse events (irAEs) (2–4). The true incidence of cardiac irAEs due to ICIs is unknown; current estimates suggest less than 1% of patients (2).

**Figure 1:**
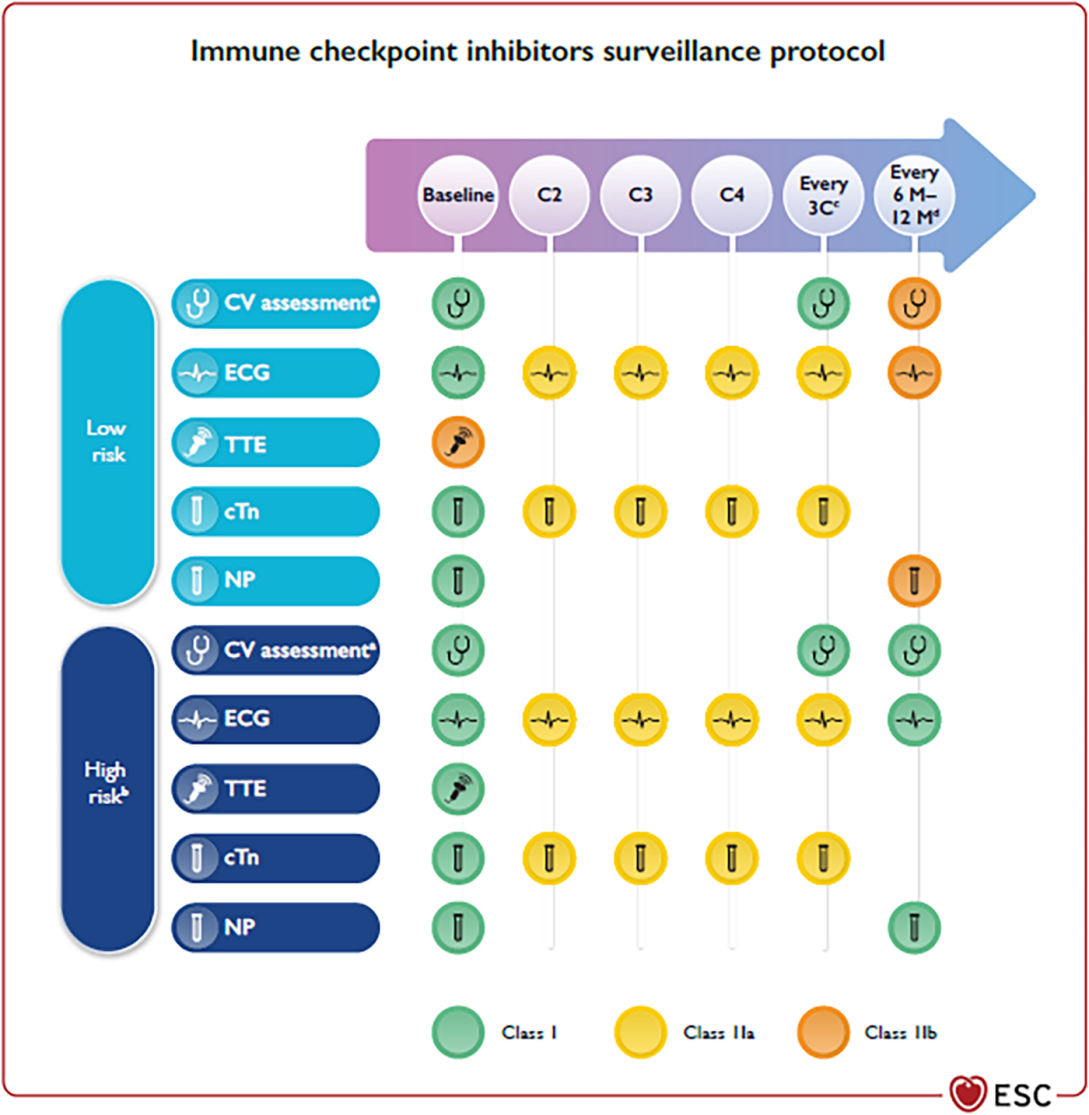
(reproduced with permission from 9). Cardiovascular surveillance in patients treated with immune checkpoint inhibitors. BNP, B-type natriuretic peptide; BP, blood pressure; C, chemotherapy cycle; cTn, cardiac troponin; CV, cardiovascular; CVD, cardiovascular disease; CTRCD, cancer therapy-related cardiac dysfunction; CTR-CVT, cancer therapy-related cardiovascular toxicity; CVRF, cardiovascular risk factors; ECG, electrocardiogram; HbA1c, glycated haemoglobin; ICI, immune checkpoint inhibitors; M, months; NP, natriuretic peptides (including BNP and NT-proBNP); NT-proBNP, N-terminal pro-B-type natriuretic peptide; TTE, transthoracic echocardiography; AF, atrial fibrillation. ^a^Including physical examination, BP, lipid profile, and HbA1c. ^b^Dual ICI, combination ICI-cardiotoxic therapy, ICI-related non-CV events, prior CTRCD or CVD. ^c^Every three cycles until completion of therapy to detect subclinical ICI-related CV toxicity. ^d^In patients who require long-term (>12 months) ICI treatment.

The largest case series of 122 subjects with ICI-associated myocarditis had early symptoms (median of 30 days after initial exposure to ICI), and up to 50% died (5). Late cardiovascular (CV) events (>90 days) are not well characterized but usually show higher risk of non-inflammatory heart failure (HF), progressive atherosclerosis, hypertension, and mortality rates (6). Other CV toxicities described during ICI therapy are MI, Atrio-Ventricular (AV) block, supraventricular and ventricular arrhythmias, sudden death, Takotsubo-like syndrome (TTS), hypercholesterolaemia, pericarditis, pericardial effusion, ischaemic stroke, and VTE (7, 8). Conditions related with high baseline ICI-related CV toxicity risk include dual ICI therapy (e.g., ipilimumab and nivolumab), combination ICI therapy with other cardiotoxic therapies, and patients with ICI-related non-CV events or prior cancer therapy-related cardiac dysfunction (CTRCD) or cardiovascular disease (CVD) (Figure 1) (9–11).

More recently, engineered T cells with chimeric antigen receptors (CAR-T cells) are being used for acute lymphocytic leukaemia and aggressive B-cell lymphomas (12). There is a growing recognition of the association between CAR-T therapy and cancer therapy-related cardiovascular toxicity (CTR-CVT), including left ventricular dysfunction (LVD), HF, cardiac arrhythmias, pericardial effusion, TTS, and cardiac arrest (13–18). The majority of the described CV toxicities have been shown to be associated with cytokine release syndrome (CRS) (12, 19), a systemic inflammatory response due to the widespread release of cytokines.

## Broad spectrum of cardiotoxicities

Acknowledging the revolutionary advances in cancer obtained with immunotherapies (and unfortunately also their toxicities), it is noteworthy that this Special Issue contains several papers that describe toxicities from immunologic therapies. Su and Colleagues (20) report a case of head and neck squamous cell carcinoma treated with pembrolizumab, a humanized monoclonal IgG4 antibody, that binds to programmed death receptor-1 (PD-1) and blocks its interaction with programmed death ligand-1 (PD-L1). The authors report that pembrolizumab-induced atrioventricular block complicated by myocarditis, successfully treated with glucocorticoids within 24 h after initial symptoms. Although left ventricular ejection fraction (LVEF) was normal, speckle tracking echocardiography revealed a slightly decreased left ventricular global longitudinal strain (GLS).

Nivolumab (anti-PD-1) and Pembrolizumab were probably the cause of immune related pericarditis, pericardial effusion and tamponade in two cases described by Chye and coworkers, who underlined the importance of monitoring and follow-up of selected patients for rechallenge with ICI after full recovery from immune-related pericardial disease (21) The two patients were suffering from advanced non-small cell lung cancer (NSCLC).

Sintilimab is a humanized monoclonal IgG4 antibody approved for the treatment of hematological cancers and several advanced solid tumors in China. Lin and collaborators presented a sintilimab related decrease of LVEF at echocardiography (22) in a patient with advanced lung adenocarcinoma. Echocardiography showed severely impaired heart function with a LVEF of 35% on admission. A significant improvement of LVEF to 52% was noted several days after treatment with methylprednisolone and immunoglobulin. However, cardiac magnetic resonance (CMR) showed extensive myocardium fibrosis. Therefore, longer follow-up is warranted to determine whether myocardial fibrosis can fully regress and to observe the long-term prognosis of the patient (Lin et al.).

Toripalimab is a PD-1 monoclonal antibody approved by the National Medical Products Administration of China in 2018. A phase I trial registered with U.S. National Library of Medicine (identifier NCT03474640) is underway in the USA. Luo and coworkers describe a case presenting with polymyositis, myocarditis, and myasthenia gravis after toripalimab used for treatment of metastatic thymoma. Toripalimab can provoke diffuse inflammation of myocytes from striated muscles to Myocardial cells, worsened in case of thymic epithelial tumors, because of the alteration of T cells immune tolerance (23).

Another manuscript shows acute pericardial effusion with cardiac tamponade with CAR-T therapy (24) in a patient with diffuse large B-cell lymphoma. Specifically, the Authors evidenced that a rapid introduction of immunosuppressive therapy can reduce the need of pericardiocentesis.

## Discussion

The conditions and toxicities described in the above-mentioned papers of this Special Issue are well recognized by the first ESC 2022 Cardio-Oncology Guidelines, which recommend that all patients on ICI treatment should have an ECG and troponin assay at baseline (Figure 1) (9). In addition, high-risk patients should undergo trans-thoracic echocardiography (TTE). Once started on therapy, ECG, cTn, and NP should be checked (9). In high-risk patients, and in those with high baseline cTn levels, TTE monitoring may be considered. Subjects with new ECG abnormalities, biomarker changes, or cardiac symptoms at any time, need to be promptly evaluated by cardio-oncologists, including a TTE for the assessment of LVEF and GLS, and CMR if myocarditis is suspected (9). Cessation of ICI treatment is recommended with ICI-associated myocarditis; patients should be admitted to hospital with continuous ECG monitoring. CV complications should be treated as per specific ESC Guidelines (HF (25), tachyarrhythmias (26), AV block (27, 28) or pericardial effusion (29)). Methylprednisolone 500–1,000 mg i.v. once daily for the first 3–5 days should be started as soon as possible (9).

Baseline CV evaluation including ECG, NP, and cTn is also recommended in patients who are to be treated with CAR-T. Baseline TTE should also be considered, especially in subjects with pre-existing cardiovascular risk factors (CVRF) and CVD. CRS should be suspected if a subject develops fever, with or without tachypnoea, tachycardia, hypotension, hypoxia, and/or other end-organ dysfunction hours to days after treatment. A high index of suspicion is necessary to diagnose CRS and to distinguish it from other conditions that occur in these settings (infections, HF, drug reactions, and PE) (9). A rise in cTn can be frequently observed in subjects with CRS and is linked to a higher risk for subsequent CV events (13). CAR-T was associated with tachyarrhythmias (atrial fibrillation, AF, the most common, followed by ventricular arrhythmias), cardiomyopathy, and pleural and pericardial diseases (16). Globally, the fatality rate of CV and pulmonary adverse events was 30.9% (30). Early cardiac evaluation in patients with cTn increase should include NP, ECG, and TTE (9). When suspected, a resting 12-lead ECG, continuous ECG monitoring, TTE, and cTn and NP are recommended. In severe cases admission to ICU is recommended because of the risk of malignant cardiac arrhythmias, circulatory collapse, and multiorgan system failure (9).

The above-mentioned surveillance strategies of acute fulminant myocarditis brought to the recognition of more subtle forms of heart inflammation, spanning from smouldering myocarditis to asymptomatic rises in serum troponin I (31–34). Hence, chronic (lasting for >12 weeks after ICI discontinuation) irAEs are increasingly detected, and can affect up to 40% of patients (35). Chronic irAEs are mostly endocrine or rheumatological, but may also affect other organs and systems (31). Extrapolating from non-ICI-associated myocarditis, it may be expected that subjects who recover from ICI-associated myocarditis would also experience chronic consequences related to residual cardiomyopathy as a long-term sequela (36). This brings to a fundamental long-term implication of irAEs, whether patients who experienced some benefit but also severe toxicities from ICI treatment should be rechallenged upon resolution of the irAE. Retrospective studies suggest that recurrence of irAEs occurs in approximately 25%–50% of patients rechallenged with ICIs (31, 37, 38). De-escalation of therapy (such as de-escalation from combination anti-PD-1–anti-CTLA4 antibodies to anti-PD-1 monotherapy) appears to be linked with a lower risk of irAE recurrence (18% in one series, 38). Determining which patients bring the highest risk of recurrent irAEs is challenging; colitis, pneumonitis and hepatitis seem to recur more frequently on rechallenge than do other irAEs, with older age also being associated with irAE recurrence (37). It is not fully understood whether a longer delay between discontinuation and rechallenge would also decrease the risk of recurrent toxicities. When evaluating the reintroduction of an ICI-based therapy, both the type and severity of the irAE, as well as the clinical need for rechallenge should be taken into account. If rechallenge is undertaken, patients should undergo close clinical and/or laboratory monitoring should in order to assess for possible irAE recurrence (31).
